# In Memoriam

**Published:** 1888-01

**Authors:** 


### In Memoriam.

### ADOLPH LIPPE, M. D.

In announcing to the homœopathic profession the sad news of the death of Dr. Adolph Lippe, we feel sure our readers will mourn the loss of a friend, a leader, and a teacher. For it is scarcely overstating the truth to say that in Dr. Lippe's death, Homœopathy loses its foremost therapeutist and its most successful prescriber. A most worthy successor of the great Hahnemann, whose teachings he so successfully followed, Dr. Adolph Lippe was the compeer of any of the great men who have made the name of the "old guard" so illustrious.

Dr. Lippe had not been in good health for some weeks; he had been suffering from rheumatic troubles and had been more or less confined to the house by these ailments during the past month or six weeks. But until Friday night no alarming symptoms had been noticed. Having exposed himself during the past few days of raw, inclement weather, a bad cold was contracted which speedily developed into a severe case of typhoid pneumonia, which medicines were powerless to check. From the first initial chill to within a few moments of death, Dr. Lippe retained consciousness and never seemed to have any hope of recovery. He said just a few hours before he died: "The medicines do no good, they only palliate." And so it seemed. During the last two days, Dr. Lippe, though so ill, was all the time throwing out hints for the treatment of his case. For instance, he would say this symptom indicates Nux mos., or this one, Natr. mur.; and so he would go through a list of remedies, pointing out with such rare skill their characteristics. But all without avail! Taken sick at 3 A. M. Saturday morning he died Monday, January 23d, at 9.45 A. M.

The funeral took place Thursday, January 26th, from his late residence, 1204 Walnut Street. The body was incased in a neat cloth-covered casket, and upon the plate was the simple inscription: "Adolph, Graf zur Lippe-Weissenfield. Born, May 11th, 1812; died, January 23d, 1888."

The funeral ceremonies were celebrated at St. John's Church, Thirteenth Street, above Chestnut. There was a large congregation present at the solemn services, many of whom were friends and former patients of the late distinguished homœopathic physician. The main altar and candlesticks were shrouded with the emblems of mourning, while the celebrant, Father Matthew F. Hand, was clad in black vestments. There were present in the chancel with the celebrant of the Solemn High Requiem Mass, Father P. R. O'Reilly, the pastor of the church, and Father E. V. Le Breton. As the cortege entered the church, Carl Wittig's Funeral March was rendered by the organist. The casket containing the remains was carried to the head of the centre aisle and placed upon a bier directly in front of the main altar, burning tapers surrounding the casket. The music was Ohnewald's Requiem Mass to the Sanctus. "Rest in the Lord," from *Elijah*, was sung as an offertory by Madame Osborne. The Agnus Dei was also sung by the same lady. After the Absolution of the Body the choir sang "Jerusalem, my happy home," and as the cortege passed out of the church Carl Wittig's Funeral March was again given.

The pall-bearers were J. G. Watmough, George Blight, Coleman Hall, H. W. Catherwood, Dr. E. J. Lee, Dr. Walter M. James, and Dr. P. P. Wells. Among those present in the congregation were Rev. Drs. Phillips Brooks, of Boston, and Charles D. Cooper, of the P. E. Church of the Holy Apostles of this city. The interment was at the Old Cathedral Cemetery.

Dr. Adolph Lippe came of an illustrious family, being a member of the old and noble German family of Lippe. He was born on the family estate of "Sée," near Gœrlitz, in Prussia. His parents were Count Ludwig and Countess Augusta zur Lippe. He was born on the 11th of May, 1812, and was therefore in his seventy-sixth year. He leaves a widow and one son to mourn his death; in Germany there survive him several brothers and sisters. On January 1st, 1885, Dr. Lippe lost his oldest son, Dr. Constantine Lippe; having two weeks previously (December, 1884) lost his only daughter. He never recovered from the severe shock of this double bereavement.

Dr. Lippe was educated at Berlin, and it was intended he should follow the legal profession, but his natural taste and talents inclining him to medicine, he came to America in 1837. He studied at the Homœopathic College at Allentown, then the only one in this country, and on the 27th of July, 1841, received his diploma at the hands of the late Dr. Constantine Hering. The Doctor first settled at Pottsville, and practiced for a time there, but subsequently established himself at Carlisle, where he remained for six years. Having distinguished himself through his treatment of the epidemics prevalent in the Cumberland Valley, he came to Philadelphia, beginning then his brilliant career in this city as a homœopathic practitioner and teacher. Dr. Lippe was remarkably successful in the practice of the healing art, seeming to be peculiarly fitted by nature for his profession. Indeed, it has fallen to the lot of few physicians to practice medicine so successfully as did Dr. Lippe His many wonderful cures during a practice of over forty-six years have won for him a grand reputation, besides giving relief and comfort to hundreds whom less skilled physicians had abandoned in despair. Dr. Lippe was indeed a born physician; he possessed to a remarkable degree the instinct of the true physician, sometimes discerning almost at a glance points which others observed not at all.

From 1863 to 1868 Dr. Lippe filled the chair of Materia Medica in the old Homœopathic Medical College of Pennsylvania, which his rare knowledge of the materia medica enabled him to do with peculiar success. Although always engaged in the busy work of a successful physician, even to within three days of his death, Dr. Lippe managed to contribute most copiously to the current literature of our school. He was the prime mover in establishing several homœopathic journals. Among them may be mentioned the late *Organon*, the *Hahnemannian Monthly*, and this journal. Even to mention by title his numerous papers would require almost a volume, so unceasing were his labors. In style he was positive, even to being dogmatic; the reason for this is readily found, it being due to the wonderful success he had had for a lifetime, in curing, or at least in relieving, all manner of sickness by a strict adherence to the Law of the Similars. Therefore, to doubt its efficiency in any case of disease was, in his eyes, almost a crime. And few would be more tolerant who had had his unique experience. He was an active or honorary member of numerous foreign and domestic societies. By the death of Dr. Lippe Homœopathy loses its most successful and its most celebrated physician, whose place will long remain vacant.

Numerous letters and telegrams have been received from Dr. Lippe's many friends, all expressive of grief and sympathy for the bereaved and of admiration for the deceased.

### RESOLUTIONS ADOPTED BY THE HAHNEMANNIAN ASSOCIATION OF PENNSYLVANIA.

The following preamble and resolutions were adopted by the Hahnemannian Association of Pennsylvania, at a special meeting called January 24th, to take appropriate action on the death of their deceased colleague, Dr. Adolph Lippe.

WHEREAS, This Association has heard with the deepest sorrow of the death of our venerable colleague and friend, Dr. Adolph Lippe; therefore be it

*Resolved,* That in the death of this veteran physician (one of the pioneers of Homœopathy in America) this Association has sustained an irreparable loss, the homœopathic school loses its ablest physician and greatest therapeutist; the public at large, its most successful practioner and wisest counselor.

*Resolved,* That by his untiring labors in the field of homœopathic materia medica, by his teaching, when a professor in the old Homœopathic College of Pennsylvania, by his unceasing contributions to the medical journals of his school, and by his example as a practitioner, Dr. Lippe did more for the development of Homœopathy in this country than any other physician, with the single exception of the late Constantine Hering.

*Resolved,* That his great industry, his sound and logical reasoning, his seeming intuitive perception of the trend of diseases, and his unexcelled ability for the analysis of drugs, were the causes of his success and placed Adolph Lippe, for many years, at the head of his profession as a physician and teacher.

*Resolved,* That his ever courteous manner and constant readiness to assist his professional brethren by his wise counsels have endeared him to his colleagues and will cause the name of Adolph Lippe to be long held in affectionate remembrance.

*Resolved,* That the members of this Association attend the funeral in a body.

*Resolved,* That a copy of these resolutions be transmitted to the family of our deceased friend and colleague; that they be also published in the *Public Ledger*, of this city, and in the medical journals.

ADOLPH FELLGER,<br>

MAHLON PRESTON,<br>

C. CARLETON SMITH,<br>

WM. JEFFERSON GUERNSEY, } *Committee.*<br>

JOHN V. ALLEN,<br>

WALTER M. JAMES,<br>

EDMUND J. LEE,

